# Associations between blood sex hormones, cognitive decline and incident dementia in community-dwelling older Australian women: a prospective cohort study

**DOI:** 10.1080/13697137.2025.2470458

**Published:** 2025-03-14

**Authors:** Farhana Sultana, Susan R. Davis, Rory S. Wolfe, John J. McNeil, Rakibul M. Islam

**Affiliations:** a School of Public Health and Preventive Medicine, Monash University, Melbourne, VIC, Australia;; b International Centre for Diarrheal Disease Research, Bangladesh (icddr,b), Dhaka, Bangladesh;; c Department of Endocrinology and Diabetes, Alfred Health, Melbourne, VIC, Australia

**Keywords:** Postmenopause, sex hormones, blood androgen, testosterone, dehydroepiandrosterone, cognitive decline, incident dementia

## Abstract

**Objective:**

Whether blood sex hormone concentrations predict cognitive decline and incident dementia in older women is uncertain. The Sex Hormones in Older Women (SHOW) study is a prospective cohort study of Australian women, aged at least 70 years, without cognitive impairment.

**Methods:**

Sex hormones were measured by liquid chromatography–tandem mass spectrometry, and comprehensive cognitive testing was performed at baseline and 3 years later.

**Results:**

Of the 6358 participants who had sex hormones measured, 4444 women (median age at baseline 74 years [Q1–Q3 71.7–77.5]) provided data for cognitive analyses. The findings were limited to a decline in executive function and verbal fluency was positively associated with the highest quartiles of estrone (odds ratio [OR] = 1.21, 95% confidence interval [CI] 1.01–1.45, *p* = 0.04) and dehydroepiandrosterone (DHEA) (OR = 1.21, 95% CI 1.01–1.45, *p* = 0.04), compared with the lowest quartiles. Estrone and DHEA were not associated with any other cognitive decline. Testosterone was not associated with cognitive decline. In an exploratory analysis, cognitive decline was not different in women who had estradiol below the limit of detection (66% of women) compared with women with measurable estradiol. Over a median 4.1 years of follow-up (22,518 person-years), 121 (2.2%) developed dementia; an incident rate of 5.3 per 1000 person-years. There were no associations between any hormone and incident dementia.

**Conclusions:**

The finding of a greater likelihood of a decline in executive function and verbal fluency in community-dwelling older women with the highest blood concentrations of DHEA and its metabolite estrone need reaffirmation and their clinical significance should be further investigated. These findings do not support use of estrogen or DHEA therapy to prevent cognitive decline in older women.

## Introduction

Estrogen receptors (ERs) and androgen receptors (ARs) are widespread throughout the brain and the latter are associated with hippocampal-related learning in animal models [1]. ERs have been reported to increase in postmenopausal female brains and ER density in the hippocampus has been associated with lower memory scores for postmenopausal women [2]. It has been suggested that estrogen, acting through its receptors, favorably influences cerebral blood flow and protects against neurological decline [2]. Additionally, in the brain, testosterone may protect against oxidative stress, serum apoptosis and the toxicity of soluble amyloid-beta through an AR-dependent mechanism [3]. Consequently, it has been proposed that postmenopausal hormone therapy may potentially prevent dementia in postmenopausal women [2,4,5].

Following menopause, the primary source of estrone and testosterone production is from dehydroepiandrosterone (DHEA) which is produced by the adrenals, circulates in nanomolar concentrations and crosses the blood–brain barrier [6]. DHEA is also synthesized *de novo* within the brain [7] and has been shown to be a neuroactive steroid, in addition to being converted to estrone, estradiol and testosterone within brain tissues [6]. It has been hypothesized that an age-related decline in brain DHEA production, and therefore potentially lower brain estrogen and testosterone availability, may contribute to age-associated neuronal dysfunction [8].

As sex hormone concentrations within the brain cannot be measured, various studies have examined whether blood sex hormone concentrations are associated with cognitive performance in postmenopausal women. Our systematic reviews of the literature provided inconclusive findings for both blood testosterone and DHEA/DHEAS and cognitive performance [9,10], with studies of testosterone mainly limited by measurement of this steroid by immunoassays that lack precision at the low levels that occur in postmenopausal women [4,11–16]. While circulating estrone is a robust proxy for estradiol concentrations in older postmenopausal women [17], most previous studies have only measured estradiol, not estrone [11–13,18,19].

This article reports the associations between blood DHEA, estrone and testosterone, measured with precision by liquid chromatography–tandem mass spectrometry (lCMS), and each of cognitive decline and incident dementia in a prospective cohort of community-dwelling Australian women aged 70 years and older, without clinical evidence of cognitive impairment at recruitment [20].

## Methods

### Study design and participants

The Sex Hormones in Older Women (SHOW) Study is a cohort sub-study of the longitudinal randomized ASPirin in Reducing Events in the Elderly (ASPREE) trial [21,22]. Between 10 March 2010 and 31 December 2014, the ASPREE trial recruited 16,703 Australian men and women aged 70 years and older through primary care across the southern Australian states of Victoria, South Australia, New South Wales, Tasmania and Australian Capital Territory. Participants were required to be free of cardiovascular disease events and dementia (score < 78 on the Modified Mini-Mental State Examination [3MS] or prior medical history of dementia) [23]. Participants with less than 5 years of life expectancy or having significant disability were excluded from the trial, as were regular users of aspirin and people with contraindications to aspirin, clinically significant anemia or uncontrolled hypertension [24]. The SHOW study comprised 6392 of the 9180 Australian women who provided biobank specimens at enrollment and consented to measurement of an array of biomarkers.

The ASPREE trial was approved by the ethics committee at each participating center. In Australia, primary ethics approval was granted by the Monash University Human Research Ethics Committee (CF07/3730–2006/745MC). The SHOW study was approved by the Monash Human Research Ethics Committee (CF16/10–2016000001) and the Alfred Hospital Human Research Ethics Committee (616/15). All of the participants provided written informed consent to contribute biological specimens to the ASPREE Healthy Ageing Biobank. The findings are reported in accordance with the Strengthening the Reporting of Observational Studies in Epidemiology (STROBE) guidelines for observational studies [25].

### Assessment of sex hormones

Non-fasting blood samples were obtained after the screening visit and plasma aliquots were stored under nitrogen vapor. Sex hormones were measured at baseline by lCMS in a single plasma sample without derivatization at the ANZAC Research Institute (Sydney, NSW, Australia) [20]. Both hormone standards and internal standards have been previously described in detail [26]. The assay limits of detection, limits of quantification, and within-run and between-run coefficients of variation were estrone (3.7 pmol/l, 11 pmol/l, 4.7%, 4.6–7.5%), estradiol (11 pmol/l, 18 pmol/l, 6.6%, 4.8–8.6%), testosterone (35 pmol/l, 0.09 nmol/l, 2.0%, 3.9–6.5%) and DHEA (0.07 nmol/l, 0.17 nmol/l, <10%, <10%) [27].

### Outcome measures

Trained staff administered the cognitive assessments at baseline, year 3 and year 5. Cognitive performance was determined using baseline and year 3 cognitive test outcomes to capture the maximal number of participants in the longitudinal analysis. The cognitive test battery included the 3MS as a measure of global cognition [28], the Hopkins Verbal learning Test – Revised (HVlT-R) for immediate and delayed recall [29], the single-letter (F) Controlled Oral Word Association Test (COWAT) for executive function and verbal fluency [30], and the Symbol Digit Modalities Test (SDMT) to measure psychomotor speed [31]. Incident dementia included all new dementia diagnoses between baseline and follow-up year 7.

### Cognitive decline

The practice or learning effect is an established phenomenon in which repeated administration of the same test in a different session results in improvement without any intervention. Consequently, the absence of a practice effect is widely considered indicative of cognitive decline [32,33]. However, to more robustly define cognitive decline, this study examined the results of all the cognitive tests in a subset of 1989 women who completed testing at baseline, year 3 and year 5. We observed that every individual test score decline between baseline and year 3 remained below the baseline at year 5. Thus, for this analysis, cognitive decline was defined as a reduction in any cognitive test score (negative score) between baseline and year 3.

### Incident dementia

Participants with a suspected dementia diagnosis (‘trigger’) were referred for further standardized cognitive and functional assessment. Dementia triggers included 3MS score < 78 [34], a drop of more than 10.15 points from the predicted score based on the individual’s baseline 3MS, after adjustment for age and education [35], a report of memory concerns or other cognitive problems to a specialist, or, as noted on the participant’s medical records, a clinician diagnosis of dementia or prescription of cholinesterase inhibitors [36]. At least 6 weeks after the initial dementia trigger, additional evaluations were performed to reduce the possibility of delirium. These included the Alzheimer’s Disease Assessment Scale – Cognitive subscale [37], Color Trails [38], lurian overlapping figures [39] and the Alzheimer Disease Cooperative Study Activities of Daily living scale, completed by the participant and, if available, study partner [40]. Along with the initial impression for cognitive change from the evaluating clinician; laboratory tests, brain imaging (computed tomography scan or magnetic resonance imaging), blood test results and clinical case notes were sought from clinical providers and hospitals for dementia assessment. This information was reviewed by the dementia adjudication committee comprising a panel of neurologists, neuropsychologists and geriatricians with expertise in dementia, blinded to treatment allocation. The date of diagnosis of dementia was taken as the date the dementia trigger occurred that resulted in a confirmed dementia diagnosis by the adjudication committee.

### Potential confounders

Baseline information included demographics (age, education, living circumstances), lifestyle factors (smoking and alcohol), body mass index and ever diagnosis of diabetes, hypertension, depression or impaired renal function. Diabetes was defined as self-reported diabetes, having a fasting plasma glucose concentration of at least 126 mg/dl (≥7 mmol/l) or receiving treatment for diabetes at baseline [41]. Hypertension was defined as blood pressure above 140/90 mmHg or treatment of hypertension at study entry. Impaired renal function was defined as an estimated glomerular filtration rate of less than 60 ml/min per 1.73 m^2^ [42].

### Statistical analysis

Categorical data are reported as percentages, and continuous data as the median (Q1–Q3) due to the skewed distribution. Women using sex hormone therapy, anti-estrogen or anti-androgen therapy, or systemic glucocorticosteroids were excluded from the analysis. For hormone results, median and interdecile ranges were reported and quartiles (lowest quartile [Q1] as the reference) were used to investigate associations with cognitive outcomes.

To explore the associations between sex hormones and cognitive decline and incident dementia, both univariable and multivariable logistic regression analyses were used and reported with the odds ratio and 95% confidence interval (CI). For longitudinal data, the delta (change score = follow-up – baseline score) values of the 3MS, Immediate HVlT-R, Delayed HVlT-R, COWAT and SDMT were used to assess cognitive decline over a 3-year period. A binary variable was computed to perform logistic regression to identify cases with accelerated cognitive decline, who may have been at risk for dementia. For this, we used decline (participants with negative cognitive scores) versus not decline (participants with no change or positive cognitive scores). An exploratory analysis was also employed to examine the association between women who had detected estradiol and who had estradiol below the limit of detection and cognitive decline.

Associations between sex hormones and incident dementia were examined by Cox proportional hazards regression analysis. Both graphical displays and testing to examine the proportional hazards assumption were used. For the proportional hazards assumption, a regression model of scaled Schoenfeld residuals against time was assessed for zero slope and all *p*-values were found to be >0.1, indicating that the proportional hazards assumptions for incident dementia were satisfactory. Unadjusted Kaplan–Meier failure curves were used to illustrate the associations between hormones and the risk of incident dementia.

Potential risk factors for cognitive decline and incident dementia included in the multivariable regression models were age, body mass index, education, smoking status, alcohol consumption, living alone, diabetes, hypertension, depression (Center for Epidemiologic Studies Depression [CESD] score >10, designated as ‘depression’) and impaired renal function. Multicollinearity between independent variables was evaluated before entering them into the model. All statistical tests were two-sided, and *p* < 0.05 was considered statistically significant. Statistical analyses were performed by Stata 17.0 (Stata Corporation, College Station, TX, USA).

## Results

**Figure 1.**
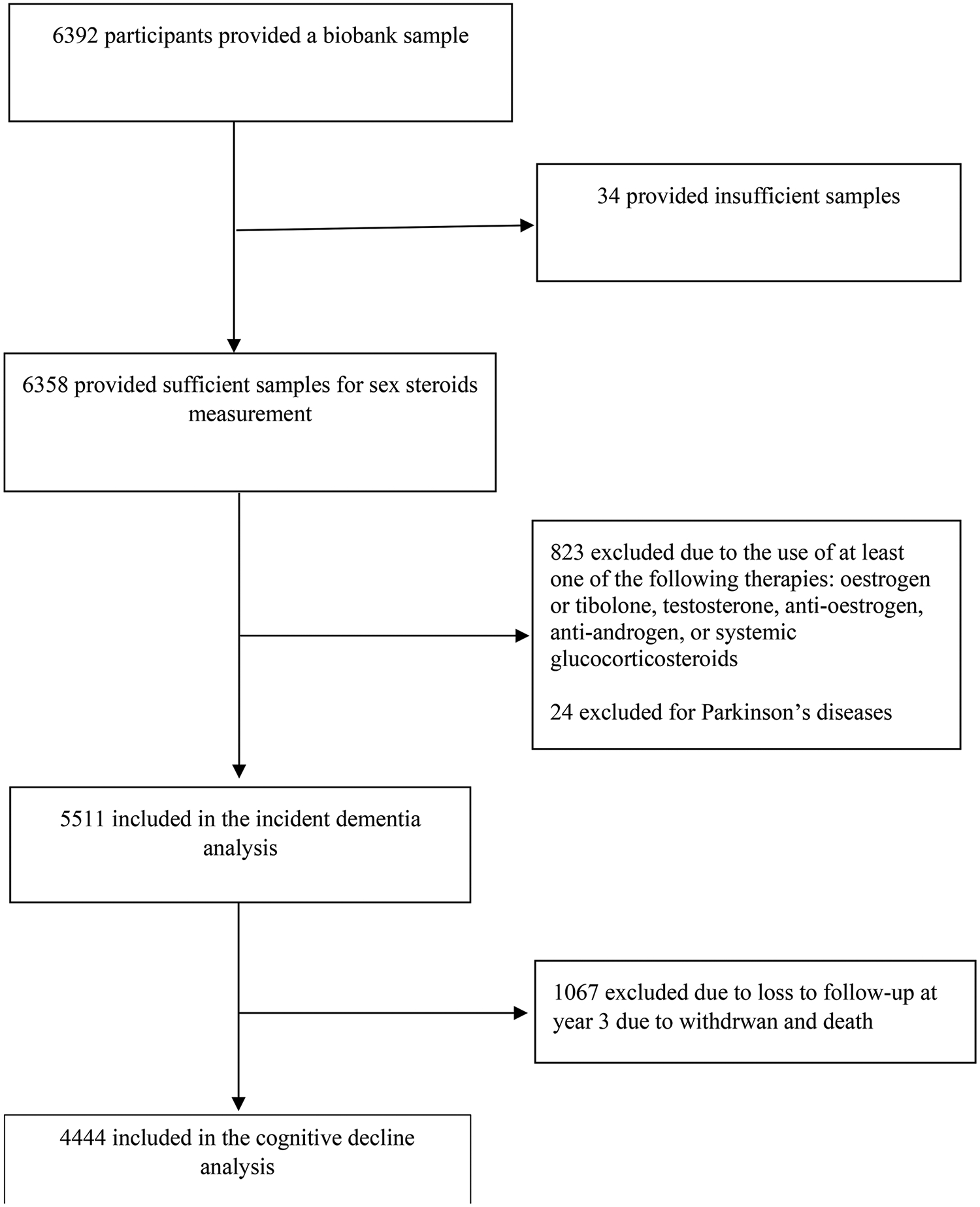
Study profile.

A total 6358 (99.5%) of the SHOW study participants provided sufficient samples from which baseline serum concentrations of blood sex hormones could be measured (Figure 1). After the exclusion of 823 participants using medications that might influence sex hormones, 24 women with Parkinson’s disease and 1067 lost to follow-up, the cognitive performance analyses included 4444 participants. Their median age at baseline was 74 years (Q1–Q3 71.7–77.5) years. The majority were overweight or had obesity (70.5%) or hypertension (73%), and 40.8% were living alone (Table 1). When compared with the included participants, the 1067 women lost to follow-up were more likely to have depression (229 [5.1%] of 4444 participants vs. 86 [8.1%] of 1067 participants; *p* ≤ 0.001) and diabetes (328 [7.2%] of 4444 participants vs. 108 [10.1%] of 1067 participants; *p* ≤ 0.01). The median age of the 5511 participants included in the analysis of incident dementia was 73.9 (Q1–Q3 71.6–77.6) years. Of these, 70.8% were overweight or had obesity, 73.2% had hypertension and 41.1% were living alone (Table 1).

Of the 4444 women who provided data for cognitive performance analysis, 3893 (88%) exhibited a decline in at least one test score at 3 years; and of these, 2601 (58%) exhibited a decline in performance in more than one test.

In the adjusted analysis, a statistically significantly greater likelihood of decline in executive function and verbal fluency performance was seen for participants with the highest estrone concentrations (Q4) compared with Q1 (Q4 vs. Q1; adjusted odds ratio [aOR] = 1.21, 95% CI 1.01–1.45, *p* = 0.04) (Table 2) and DHEA concentrations (Q4 vs. Q1; aOR = 1.21, 95% CI 1.01–1.45, *p* = 0.04). Having the highest testosterone concentrations (Q4) compared with Q1 approached significance for an association with a decline in executive function and verbal fluency (Q4 vs. Q1; aOR = 1.19, 95% CI 0.99–1.42, *p* = 0.06). Estrone and testosterone concentrations were not associated with a decline in performance on any other cognitive test. A lower likelihood of a decline in immediate HVlT-R was seen for participants with DHEA concentrations in Q2 compared with Q1 in the adjusted analysis (aOR = 0.81, 95% CI 0.67–0.96, *p* = 0.02).

In the exploratory analysis, cognitive decline was not different in 66% of women with estradiol concentrations below the limit of detection compared with those with measurable estradiol (Table 3).

**Figure 2.**
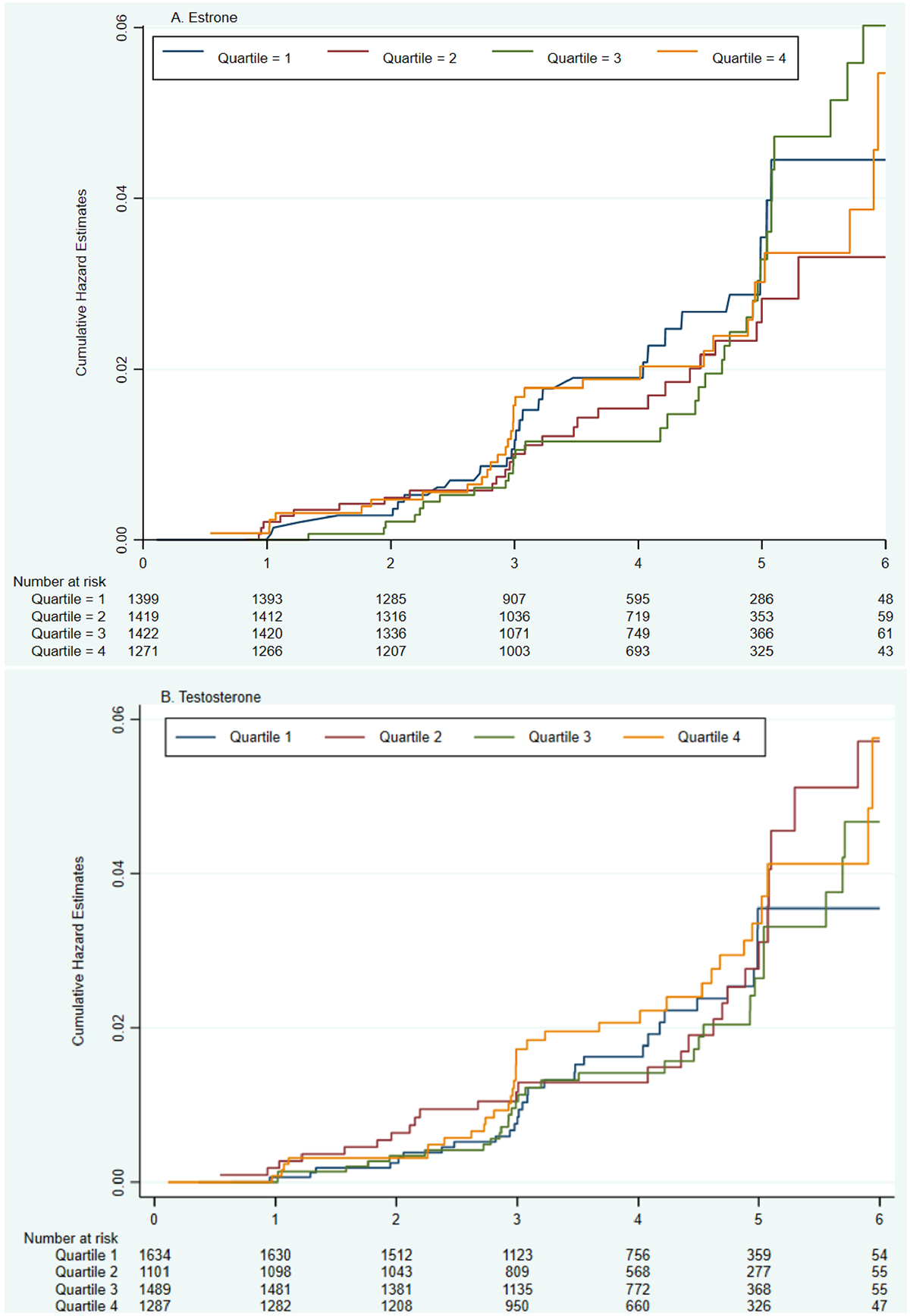

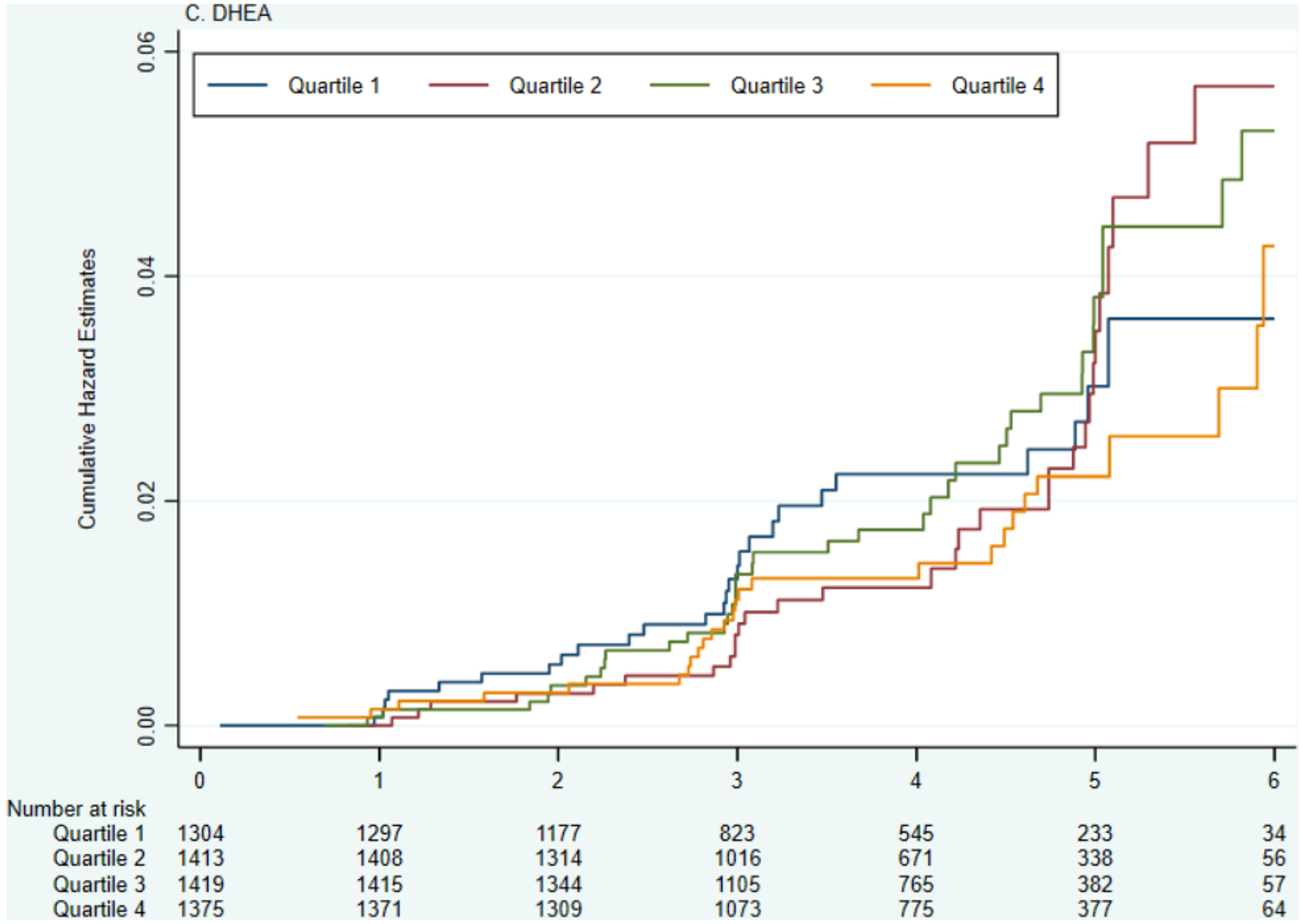
Cumulative hazard estimates for incident dementia by quartiles of androgens in the blood: (A) estrone, (B) testosterone and (C) dehydroepiandrosterone (DHEA)

Over a median of 4.1 years (Q1–Q3 2.9–4.9) of follow-up (22,518 person-years), 121 (2.2%) of the 5511 women developed incident dementia; an incident rate of 5.3 per 1000 person-years. The cumulative hazards of incident dementia did not differ by estrone, testosterone or DHEA concentration quartiles (Figure 2). With Q1 as the reference, the risk of incident dementia by sex hormone quartiles were examined with and without adjustment for risk factors for dementia (Table 4). There were no associations between any hormone and incident dementia.

## Discussion

This study has shown limited associations between blood sex steroid concentrations and cognitive change in community-dwelling women, aged at least 70 years and cognitively unimpaired at baseline. Specifically, blood estrone and DHEA concentrations in the highest quartiles were associated with a greater likelihood of a decline in verbal fluency and executive function, with testosterone approaching statistical significance, over 3 years of follow-up. A lower likelihood of a decline in immediate recall in women with DHEA concentrations in Q2 was an isolated finding.

We were specifically interested in exploring the associations between estrone and testosterone and cognitive function in women aged 70 years and older, having found that concentrations of these hormones from the age of 70 years were not only similar to concentrations in healthy premenopausal women [20,43] but also increased with age [20,44]. The study subsequently found that low blood testosterone and DHEA concentrations were associated with a two-fold greater likelihood of a major ischemic cardiovascular event in this population [44]. Hence, given that sex hormones have been implicated as protecting against Alzheimer’s disease [2] together with vascular cognitive decline being the leading cause of dementia after Alzheimer’s disease [45], our expectation was that low blood sex hormone concentrations would be associated with a greater likelihood of cognitive decline.

The consistent association between estrone, and its precursor DHEA, and the decline in COWAT scores suggests these findings may be biologically significant. However, this finding was unexpected, and potentially a chance finding. Whether these sex hormones have physiologically important roles in cognitive decline should be considered uncertain, and any explanation of our findings would be highly speculative. This is because sex steroid action is tissue-specific and modulated by intracellular and membrane receptor availability and binding, which is in turn modulated by coactivators and co-repressors, transcription factors and other neurosteroid concentrations [46]. Nonetheless, our overall findings do not support the use of sex hormone therapy to prevent cognitive decline in older women.

There are few studies with which to directly compare our findings. Kische et al. reported no association between the change in global cognition, assessed by the 30-item Mini-Mental State Examination (MMSE), and testosterone measured by lCMS in a community-based sample of women aged 20–80 years [47]. The Rancho Bernardo Study found that higher levels of endogenous estrone, measured by immunoassays in blood samples collected an average of 4 years before the baseline cognitive assessment, were associated with a greater 4-year decline in verbal fluency in the sample of community-dwelling individuals aged 70 years [48]. Another study by Koyama et al. reported no association between estrone, testosterone and DHEA and cognitive function, assessed 4 years and 9.5 years after blood was drawn, for women aged 70 and older [19]. However, baseline testing was not performed, change was not reported and the hormone measurement method was not specified. Our recent systematic review [10] and another systematic review by Boss et al. demonstrated uncertain prospective associations between sex hormones and cognitive performance in postmenopausal women due to methodological issues including failure to consider users of medications that might influence blood hormone concentrations, and differences in study designs, samples and in the tests used to assess cognitive function [49].

Our study strengths are the large community-based sample of cognitively unimpaired women aged 70 years and older, measurement of hormones by lCMS, formal testing of cognitive performance using a comprehensive neuropsychological test battery and rigorous exclusion of women taking medications potentially influencing blood hormone concentrations. As hormone action is not necessarily linear [50], the use of multiple logistic regression models minimized the potential heterogeneity of measurement bias. We defined a decline in cognitive performance as a decline in the cognitive test score from each individual’s baseline. In contrast, other studies have defined significant cognitive decline as >1.0, 1.5 or 2.0 standard deviations of the mean cognitive score for a specific cognitive test [36,51,52]. This approach does not take into account the significance of the absence of a practice effect decline [32,33,53]. Furthermore, variations in the operational criteria, such as application of different cut-off scores, can inflate or underestimate prevalence [54] and reduce specificity [55–57]. We believe our approached captured all of the participants who demonstrated a persistent decline and did not misclassify any of the participants who actually declined as ‘not declined’. As our sample comprised predominantly of women of European ancestry, our findings cannot be generalized to women of other ancestries.

## Conclusions

In summary, the highest quartiles of blood estrone and DHEA were associated with a greater likelihood of decline in verbal fluency and executive function (COWAT) with testosterone not quite reaching statistical significance over 3 years of follow-up in community-dwelling older Australian women without cognitive impairment at baseline. The consistent finding for all three sex steroids suggests this may be biologically meaningful and requires reaffirmation in other populations. Any explanation for our findings is highly speculative. However, our findings do prompt questions with regard to initiating the use of estrogen or hormone therapy in older women.

## Figures and Tables

**Table 1.** Characteristics of women at baseline, and differences between women followed up at year 3 and those lost to follow-up.

		Difference		
Characteristic	Baseline (*n* = 5511)	Follow-up 3 (n = 4444)	Lost to follow-up (n = 1067)	*p*-Value
Age (years), median (IQR)	73.9 (71.6–77.6)	74.0 (71.7–77.5)	73.8 (71.5–70.2)	0.75
Age group, *n* (%)				
70–74	2766 (50.2)	2218 (49.9)	548 (51.4)	<0.01
75–79	1739 (31.6)	1445 (32.5)	294 (27.5)	
80–84	758 (13.7)	594 (13.4)	164 (15.4)	
≥85	248 (4.5)	187 (4.2)	61 (5.7)	
Weight (kg), median (IQR)^a^	69.8 (61.8–79.2)	69.6 (61.8–79)	70.3 (61.9–79.8)	0.24
Height (m), mean (SD)^b^	1.59 (± 0.06)	1.59 (± 0.06)	1.59 (± 0.06)	0.95
Body mass index (kg/m^2^), *n* (%)^c^				
<18.5	44 (0.8)	32 (0.7)	12 (1.1)	0.27
18.5–24.9	1557 (28.4)	1272 (28.6)	285 (26.7)	
25.0–29.9	2173 (39.6)	1757 (39.5)	416 (38.9)	
≥30.0	1708 (31.2)	1361 (30.6)	347 (32.5)	
Years of education, *n* (%)				
<12	2867 (52.0)	2314 (52)	549 (51.4)	0.68
≥12	2644 (48.0)	2126 (47.8)	518 (48.5)	
Depression, *n* (%)	315 (5.7)	229 (5.1)	86 (8.1)	<0.001
Diabetes, *n* (%)	436 (7.9)	328 (7.2)	108 (10.1)	<0.01
Hypertension, *n* (%)	4037 (73.2)	3244 (73)	793 (74.3)	0.38
Living alone, *n* (%)	2267 (41.1)	1815 (40.8)	452 (42.4)	0.29
Smoking status, *n* (%)				
Current	156 (2.8)	112 (2.5)	44 (4.1)	<0.001
Former	1735 (31.5)	1365 (30.7)	370 (34.7)	
Never	3620 (65.7)	2967 (66.8)	653 (61.2)	
Alcohol consumption, *n* (%)				
Current	4114 (74.6)	3344 (75.2)	770 (72.2)	<0.01
Former	215 (4.0)	158 (3.5)	57 (5.3)	
Never	1182 (21.4)	942 (21.2)	240 (22.5)	
Impaired renal function, *n* (%)^d^	1004 (18.7)	804 (18.1)	200 (18.7)	0.69
Blood sex hormones, median (10th–90th centile)				
Estrone (pmol/l)	181.2 (85.0–343.9)	181.2 (88.8–347.7)	173.8 (81.4–340.3)	<0.01
Testosterone (nmol/l)	0.38 (0.17–0.85)	0.38 (0.17–0.87)	0.35 (0.13–0.93)	0.35
DHEA (nmol/l)	2.60 (1.04–6.10)	2.63 (1.04–6.10)	2.46 (0.83–6.10)	<0.01

a Data available: weight (*n* = 4426).

b Data available: height (*n* = 4437).

c Data available: body mass index (*n* = 4422).

d Data available: impaired renal function (*n* = 4315).

DHEA, dehydroepiandrosterone; IQR, interquartile range; SD, standard deviation.

**Table 2.** Associations between estrone, testosterone and DHEA and cognitive decline.

Blood sex hormones, *N* (%), median (10th–90th centile)	3MS (global cognition)	Immediate HVLT-R	Delayed HVLT-R	COWAT (executive function and verbal fluency)	SDMT (psychomotor speed)
Estrone					
Quartile 1: 1166 (26.2), 99.9 pmol/l (48.1–125.7)	1 (ref.)	1 (ref.)	1 (ref.)	1 (ref.)	1 (ref.)
Quartile 2: 1080 (24.3), 155.3 pmol/l (136.8–177.5)	1.01	1.02	1.09	0.99	0.92
	(0.85–1.21)	(0.85–1.22)	(0.91–1.31)	(0.83–1.19)	(0.78–1.09)
	*p* = 0.87	*p* = 0.84	*p* = 0.35	*p* = 0.93	*p* = 0.36
Quartile 3: 1101 (24.7), 218.2 pmol/l (188.6–255.2)	0.99	0.95	0.99	0.94	1.11
	(0.84–1.19)	(0.79–1.14)	(0.82–1.19)	(0.79–1.13)	(0.94–1.32)
	*p* = 0.98	*p* = 0.60	*p* = 0.94	*p* = 0.54	*p* = 0.21
Quartile 4: 1097 (24.7), 329.2 pmol/l (277.4–484.5)	1.11	1.01	0.97	1.21	0.93
	(0.93–1.32)	(0.84–1.21)	(0.81–1.18)	(1.01–1.45)	(0.78–1.11)
	*p* = 0.26	*p* = 0.97	*p* = 0.79	*p* = 0.04	*p* = 0.43
Testosterone					
Quartile 1: 1297 (29.2), 0.173 nmol/l (0.104–0.243)	1 (ref.)	1 (ref.)	1 (ref.)	1 (ref.)	1 (ref.)
Quartile 2: 1155 (26.0), 0.312 nmol/l (0.277–0.381)	1.03	0.84	0.91	1.16	1.02
	(0.87–1.22)	(0.72–1.01)	(0.76–1.09)	(0.99–1.38)	(0.86–1.20)
	*p* = 0.69	*p* = 0.06	*p* = 0.31	*p* = 0.09	*p* = 0.83
Quartile 3: 950 (21.4), 0.451 nmol/l (0.416–0.555)	0.99	0.92	0.95	1.09	0.99
	(0.83–1.19)	(0.77–1.11)	(0.79–1.15)	(0.91–1.32)	(0.83–1.18)
	*p* = 0.98	*p* = 0.40	*p* = 0.59	*p* = 0.32	*p* = 0.91
Quartile 4: 1042 (23.4), 0.797 nmol/l (0.589–1.525)	1.03	0.96	0.92	1.19	0.914
	(0.87–1.23)	(0.81–1.15)	(0.77–1.11)	(0.99–1.42)	(0.77–1.08)
	*p* = 0.72	*p* = 0.67	*p* = 0.40	*p* = 0.06	*p* = 0.29
DHEA					
Quartile 1: 1117 (25.1), 1.179 nmol/l (0.555–1.595)	1 (ref.)	1 (ref.)	1 (ref.)	1 (ref.)	1 (ref.)
Quartile 2: 1114 (25.1), 2.184 nmol/l (1.768–2.565)	0.86	0.81	0.92	1.12	1.03
	(0.72–1.02)	(0.67–0.96)	(0.77–1.11)	(0.93–1.34)	(0.87–1.23)
	*p* = 0.09	*p* = 0.02	*p* = 0.40	*p* = 0.23	*p* = 0.70
Quartile 3: 1108 (25.0), 3.258 nmol/l (2.774–3.848)	0.92	0.87	1.01	1.02	1.08
	(0.77–1.09)	(0.73–1.04)	(0.83–1.21)	(0.85–1.23)	(0.91–1.29)
	*p* = 0.36	*p* = 0.14	*p* = 0.93	*p* = 0.83	*p* = 0.37
Quartile 4: 1105 (24.8), 5.582 nmol/l (4.229–8.598)	0.97	0.99	1.01	1.21	1.03
	(0.82–1.16)	(0.82–1.19)	(0.84–1.22)	(1.01–1.45)	(0.86–1.22)
	*p* = 0.77	*p* = 0.93	*p* = 0.89	*p* = 0.04	*p* = 0.75

Data presented as adjusted odds ratio (95% confidence interval): models are adjusted for age, body mass index, education, depression, diabetes, hypertension, living alone, smoking status, alcohol consumption and impaired renal function. COWAT, Controlled Oral Word Association Test; DHEA, Dehydroepiandrosterone; HVlT-R, Hopkins Verbal learning Test – Revised; 3MS, Modified Mini-Mental State Examination; SDMT, Symbol Digit Modalities Test.

**Table 3.** Association between estradiol (detected versus not detected) and cognitive decline.

Estradiol, *N* (%), median (10th–90th centile)	Global cognition (3MS)	Immediate recall (HVLT-R)	Delayed recall (HVLT-R)	Executive function and verbal fluency (COWAT)	Psychomotor speed (SDMT)
Detected: 1464 (32.9), 22.0 pmol/l (11.0–58.7)	1 (ref.)	1 (ref.)	1 (ref.)	1 (ref.)	1 (ref.)
Not detected: 2980 (67.1)	0.96	1.06	0.98	0.91	0.99
	(0.84–1.10)	(0.93–1.22)	(0.85–1.13)	(0.79–1.05)	(0.87–1.13)
	*p* = 0.60	*p* = 0.38	*p* = 0.82	*p* = 0.19	*p* = 0.88

Data presented as adjusted odds ratio (95% confidence interval): models are adjusted for age, body mass index, education, depression, diabetes, hypertension, living alone, smoking status, alcohol consumption and impaired renal function. COWAT, Controlled Oral Word Association Test; HVlT-R, Hopkins Verbal learning Test – Revised; 3MS, Modified Mini-Mental State Examination; SDMT, Symbol Digit Modalities Test.

**Table 4.** Associations between estrone, testosterone and DHEA and incident dementia.

Sex hormone median blood concentration, *N* (%), median (10th–90th centile)	Incident dementia
Estrone	
Quartile 1: 1399 (25.4), 96.2 pmol/l (48.1–122.0)	1 (ref.)
Quartile 2: 1419 (25.7), 155.3 pmol/l (133.1–177.5)	0.83 (0.48–1.42), *p* = 0.49
Quartile 3: 1422 (25.8), 220.1 pmol/l (188.6–258.9)	0.99 (0.50–1.66), *p* = 0.99
Quartile 4: 1271 (23.1), 336.6 pmol/l (284.8–484.5)	0.97 (0.58–1.64), *p* = 0.91
Testosterone	
Quartile 1: 1634 (29.6), 0.17 nmol/L (0.10–0.24)	1 (ref.)
Quartile 2: 1101 (20.0), 0.31 nmol/L (0.28–0.35)	1.22 (0.72–2.08), *p* = 0.46
Quartile 3: 1489 (27.0), 0.45 nmol/L (0.38–0.55)	1.07 (0.64–1.79), *p* = 0.78
Quartile 4: 1287 (23.4), 0.79 nmol/L (0.59–1.9)	1.27 (0.77–2.09), *p* = 0.36
DHEA	
Quartile 1: 1304 (23.7), 1.11 nmol/L (0.52–1.52)	1 (ref.)
Quartile 2: 1413 (25.6), 2.08 nmol/L (1.69–2.46)	1.08 (0.64–1.84), *p* = 0.77
Quartile 3: 1419 (25.7), 3.19 nmol/L (2.70–3.81)	1.37 (0.82–2.28), *p* = 0.22
Quartile 4: 1375 (25.0), 5.58 nmol/L (4.19–8.63)	1.12 (0.64–1.95), *p* = 0.69

Data presented as adjusted hazard ratio (95% confidence interval): models are adjusted for age, body mass index, education, depression, diabetes, hypertension, living alone, smoking status, alcohol consumption and impaired renal function. DHEA, dehydroepiandrosterone.

## Data Availability

After deidentification (i.e. text, tables, figures and supplementary material), individual participant data will be made available. On application, meta-data and a data dictionary will be made available to others. The ASPirin in Reducing Events in the Elderly (ASPREE) study protocol is available on the ASPREE website. The ASPREE trial statistical analysis plan has been published [58]. On request, a copy of the clinical trial consent form can be made available. Requests for data access will be via the ASPREE Principal Investigators with details for applications provided through the Sex Hormones in Older Women (SHOW) study Principal Investigator. Sub-study data on sex hormones can be requested through this system with approval by the corresponding author. Data will be made available to investigators whose proposed use of the data, registered as a project through the ASPREE Access Management Site, has been approved by a review committee. Access will be through a secure web-based data portal (the ASPREE Safe Haven system), based at Monash University (Monash, VIC, Australia).
